# Multifaceted Roles of the *N*^6^-Methyladenosine RNA Methyltransferase METTL3 in Cancer and Immune Microenvironment

**DOI:** 10.3390/biom12081042

**Published:** 2022-07-28

**Authors:** Chenxi Hu, Jiacheng Liu, Yue Li, Wei Jiang, Ding Ji, Wei Liu, Teng Ma

**Affiliations:** 1 Department of Immunology, School of Basic Medicine, Hebei Medical University, 361 Zhongshan East Rd., Shijiazhuang 050017, China; chenxi.h@outlook.com (C.H.); ljc15632502918@163.com (J.L.); liyue1003141265@163.com (Y.L.); a18031704335@163.com (W.J.); ding-j@hotmail.com (D.J.); 2 The First Hospital of Hebei Medical University, Shijiazhuang 050030, China; 3 The Forth Hospital of Hebei Medical University, Shijiazhuang 050011, China; 4 Cancer Research Center, Beijing Chest Hospital, Capital Medical University/Beijing Tuberculosis and Thoracic Tumor Research Institute, Beijing 101149, China

**Keywords:** METTL3, methyltransferase, m^6^A, RNA modification, tumor immune microenvironment

## Abstract

As the most abundant internal mRNA modification in eukaryotic cells, *N*^6^-methyladenosine (m^6^A) has emerged as an important regulator of gene expression and has a profound impact on cancer initiation and progression. mRNA m^6^A modification is regulated by m^6^A methyltransferases, demethylases and reader proteins to fine tune gene expression at the post-transcriptional level. The most well-studied m^6^A methyltransferase, METTL3, plays critical roles in regulating gene expression and affecting the outcome of various cancers. In this review, we discuss the multifaceted roles of METTL3 in regulating specific molecular signaling pathways in different types of cancers and the recent progress on how METTL3 impacts the tumor immune microenvironment. Finally, we discuss future directions and the potential for therapeutic targeting of METTL3 in cancer treatment.

## 1. Introduction

To date, more than 100 chemical modifications on RNA have been identified in all kingdoms of life [1]. Among these modifications, *N*^6^-methyladenosine (m^6^A) is one of the most abundant internal modifications in eukaryotic messenger and noncoding RNAs and plays important roles in regulating RNA metabolism, such as pre-mRNA splicing [2], mRNA nuclear export [3], mRNA stability [4], mRNA translation [5], miRNA maturation [6], and long noncoding RNA (lncRNA) processing [7]. By selectively ‘marking’ mRNA with m^6^A, the cell can control gene expression, which broadly affects many processes, including cancer progression [8,9], stress response [10], stem cell differentiation [11,12], and gametogenesis [13].

*N*^6^-methyladenosine is catalyzed by a large methyltransferase complex which is localized in the nucleus. The majority of research has focused on two main subunits of this complex—methyltransferase-like 3 (METTL3) and methyltransferase-like 14 (METTL14) [14,15]. METTL3 is the sole subunit capable of independently catalyzing the deposition of m^6^A on mRNA, but direct protein–protein interactions with METTL14 significantly improve catalytic efficiency compared to METTL3 alone.

Other subunits in the m^6^A methyltransferase complex, such as Wilm’s Tumor 1-Associating Protein (WTAP) [16], RNA Binding Motif Protein 15 (RBM15) [7], Zinc finger CCCH domain-containing protein 13 (ZC3H13) [17] and VIRMA (KIAA1429) [18], play regulatory roles that confer specificity for different target mRNAs or lncRNAs. Since m^6^A is involved in pre-mRNA splicing and miRNA maturation, the deposition of m^6^A on nascent RNA transcripts must occur rapidly. Indeed, m^6^A methylation of pre-mRNA and the binding of m^6^A-binding proteins such as hnRNPG on newly methylated transcripts occurs co-transcriptionally and before transcription termination [19,20]. There are reports that METTL3 can be bound to chromatin by interacting with histone modifications such as the transcription factor CEBPZ, setting the stage for rapid m^6^A methylation of certain actively transcribed genes [8].

**Figure 1:**
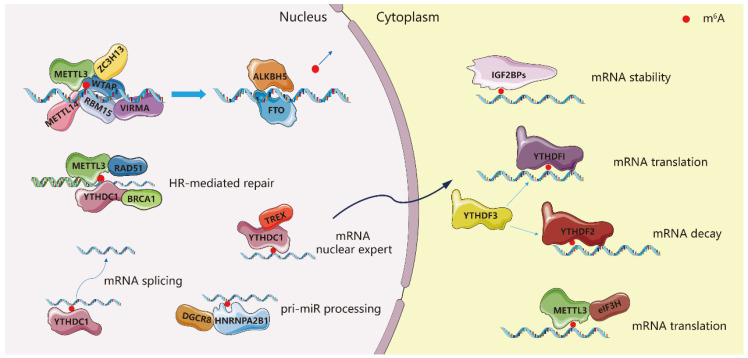
METTL3-dependent biological functions in cells.

Demethylases remove m^6^A modification from RNA, such as FTO and ALKBH5 [21,22]. The fate of methylated mRNA is decided by the binding of m^6^A-binding proteins such as YTH domain-containing proteins (YTHDF1, YTHDF2, YTHDF3, YTHDC1, and YTHDC2) [23,24,25], insulin-like growth factor 2 mRNA binding proteins (IGF2BP1 and IGF2BP2) and others [26]. Some readers affect the stability of methylated transcripts in the cytoplasm whereas others may affect splicing, nuclear export and miRNA processing [2,3,4]. Splicing of pre-mRNA can be promoted by FTO, YTHDC1 and HNRNPA2B1 [6,27,28]. To facilitate nuclear export of mRNA, the nuclear export complex TREX is recruited by the m^6^A methyltransferase complex and TREX also stimulates the recruitment of YTHDC1 to mRNA, which in turn can recruit SR proteins to promote export of m^6^A-modified mRNA [3,29]. METTL3 itself can facilitate mRNA translation by recruiting eIF3H to initiate translation in a manner independent of its catalytic activity [30] (Figure 1). In summary, throughout the various stages of pre-mRNA processing and pri-miRNA maturation, m^6^A modification is dynamically added, removed, and read by various enzymes responding to the present needs of the cell.

Furthermore, METTL3 is intimately involved in DNA damage repair [31]. Ultraviolet-induced cyclobutane pyrimidine and abasic sites with complete sugar-phosphate backbones are two important frequent DNA damages. Ultraviolet causes strand breaks, while abasic sites can lead to mutations and strand breaks [32]. The METTL3-METTL14 complex is recruited at the DNA-damaged sites to modulate nucleotide-excision repair and homologous recombination (HR)-mediated repair by recruiting RAD51 and BRCA1 (Figure 1). Mechanistically, the METTL3-METTL14 complex is active on unpaired DNA [33]; METTL3 is phosphorylated at S43 (serine phosphorylation sites) by ATM (ATM is a critical master regulator of DNA damage response signaling) and localized to double-strand breaks (DSBs) sites [34].

**Figure 2:**
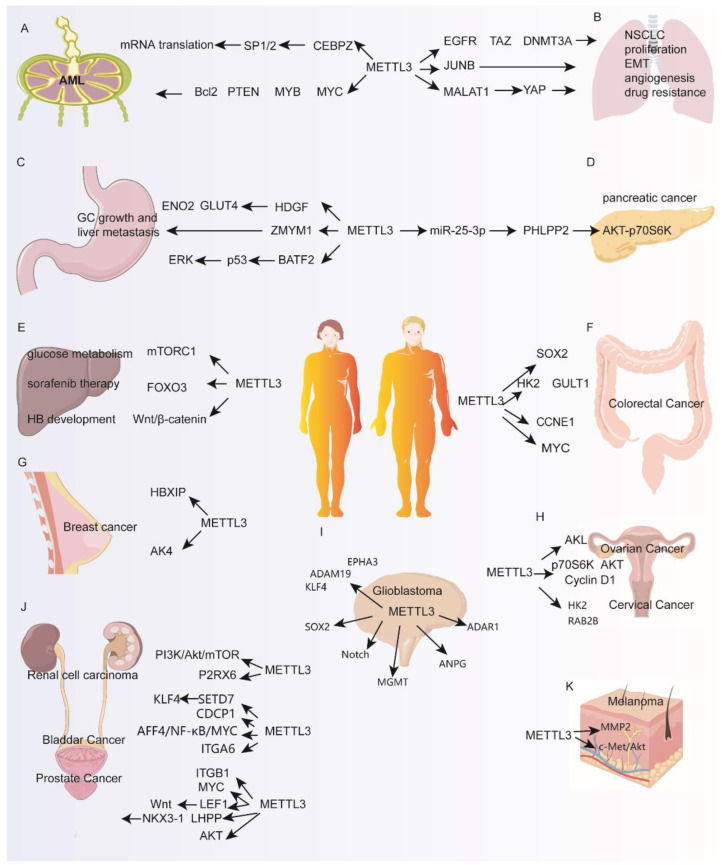
Multifaceted functions of METTL3 in different types of cancer. (**A**–**K**) propose how METTL3 regulates various genes and molecular pathways. AML, Acute myeloid leukemia; NSCLC, non-small-cell lung carcinoma; GC, Gastric cancer.

This review compiles and discusses the current literature regarding the diverse functions of METTL3 in cancer. It will also illuminate how METTL3 regulates the expression of genes and pathways in various cancers (Figure 2), which may be useful in identifying novel therapies for cancer.

## 2. Multiple Roles of the METTL3 in Cancers

### 2.1. Acute Myeloid Leukemia (AML)

AML is a malignant cancer of myeloid hematopoietic stem or progenitor cells [35]. In normal human hematopoietic stem/progenitor cells (HSPCs), METTL3 inhibits differentiation and promotes proliferation, thus playing a crucial role in normal myeloid differentiation. AML cells have higher expression of METTL3 compared to HSPCs and a CRISPR dropout screen identified METTL3, METTL14, WTAP and KIAA1429 as essential for the survival of AML cells, indicating a potential oncogenic role for m^6^A modification in AML [36]. Indeed, genetic depletion of METTL3 in AML cell lines and primary leukemic blasts lead to cell growth inhibition, cell cycle arrest, induction of apoptosis and differentiation [8,36]. Mechanistically, METTL3’s oncogenic role in AML was linked to enhancing translation of anti-apoptotic and pro-growth transcripts such as c-MYC, MYB, PTEN and BCL2 in an m^6^A-dependent manner [36]. METTL3, but not METTL14, was also found to associate with chromatin at transcription initiating sites in a CEBPZ-dependent manner [8]. Promoter-bound METTL3 methylates the coding region of target mRNAs such as SP1 and SP2, leading to an increase in translation efficiency of these mRNAs, thus promoting AML cell survival [8] (Figure 2A). Taken together, AML cells expressing a higher abundance of METTL3 compared to HSPCs can utilize m^6^A modification to promote the expression of oncogenic pathways, thus promoting leukemia.

### 2.2. Lung Cancer

Lung cancer is one of the most prevalent malignant tumors, contributing to a quarter of all cancer deaths in the USA in 2020 [37]. In lung cancer, METTL3 but not METTL14 is significantly elevated in tumors compared to normal tissue [30]. Genetic ablation of METTL3 expression in A549 cells significantly reduces cell proliferation and survival. METTL3 exerts its oncogenic role in lung cancer by enhancing the translation of epidermal growth factor receptor (EGFR), transcriptional co-activator with PDZ-binding motif (TAZ) and DNA methyltransferase 3A (DNMT3A) in a methyltransferase- or m^6^A binding protein-independent manner by recruiting the translation initiation factor eIF3h to METTL3 bound transcripts [30]. In non-small-cell lung carcinoma (NSCLC), the expression of METTL3 was negatively correlated with miR-33a expression in 32 patient-derived tissues and showed that METTL3 is a potential target of miR-33a [38]. Overexpression of miR-33a in A549 or NCI-H460 cells led to decreased METTL3 expression at both the RNA and protein levels and decreased cell proliferation [38]. Furthermore, METTL3 promotes NSCLC apoptosis, migration and invasion by mediating mature miR-1246, which targets paternally expressed gene 3 (PEG3) [39]. METTL3 promotes metastasis-associated lung adenocarcinoma transcript 1 (MALAT1) translation by recruiting YTHDF1/3. MALAT1 combines miR-1914-3p to increase yes association protein (YAP), inducing NSCLC metastasis and drug resistance [40]. Treatment of A549 and LC2/ad lung cancer cells with TGF-β caused upregulation of METTL3 and promoted epithelial to mesenchymal transition (EMT) [41]. Knockdown of METTL3 decreased m^6^A modification and mRNA stability of JUNB, one of the important transcription regulators of EMT, and also led to the expression changes in EMT-related genes such as E-cadherin, fibronectin and vimentin [41]. miR-143-3p/VASH1 was related to poor survival outcomes in lung cancer patients [42]. METTL3 elevates miR-143-3p expression by enhancing splicing of miR-143-3p to generate mature miRNA [42]. In turn, miR-143-3p can promote invasion in an in vitro blood–brain barrier model and inhibit vasohibin-1 (VASH1) expression to increase angiogenesis in lung cancer tissue [42]. Other studies found that METTL3 elevated the m^6^A level of Zinc finger and BTB domain-containing 4 (ZBTB4) mRNA in cigarette smoke extract-induced transformed HBE (T-HBE) cells. Thus, ZBTB4, a transcriptional repressor, was dependent on YTHDF2. Lower expression of ZBTB4 upregulated EZH2, which enhanced H3K27me3 combining with E-cadherin promoter, causing lower E-cadherin levels and EMT and T-HBE cell malignancy [43] (Figure 2B).

### 2.3. Gastric Cancer (GC)

METTL3 is upregulated in GC tumor cells and is an independent prognostic factor of poor survival and an effective predictor for severity of GC [44,45]. The suppression of METTL3 expression inhibits cell proliferation, migration and invasion of SGC7901 and AGS cells [44]. P300 was found to mediate H3K27 acetylation of the METTL3 promoter, thus upregulating METTL3 transcription. One of METTL3’s targets in GC cells, HDGF, is methylated by METTL3 and stabilized by the m^6^A reader IGF2BP3 [46]. HDGF can accelerate tumor angiogenesis and enhance glycolysis by increasing the expression of GLUT4 and ENO2 [46]. METTL3 also promoted liver metastasis in a mouse model of gastric cancer [46]. Another study demonstrated METTL3’s ability to facilitate EMT in vitro and promote metastasis in vivo [47]. Zinc finger MYM type containing 1 (ZMYM1) was identified as the target of METTL3 for m^6^A modification in GC cells [47]. Mechanistically, METTL3 methylates ZMYM1 mRNA and is stabilized by Human antigen R (HuR), which acts as a m^6^A “reader” protein located in the nucleus [47]. HuR is also well acknowledged as a general RNA binding protein [48]. In turn, ZMYM1 promotes EMT and metastasis by recruiting the CtBP/LSD1/CoREST complex to the E-cadherin promoter to repress its transcription. Importantly, this study reveals that the METTL3/ZMYM1/E-cadherin signaling pathway is a potential therapeutic target for GC metastasis [47]. Another study showed that BATF2 increases p53 protein stability, inhibiting the phosphorylation of extracellular signal-regulated kinase (ERK). METTL3 represses BATF2 expression, thus promoting GC development [49]. Silencing hepatitis B X-interacting protein (HBXIP) causes low METTL3 expression. METTL3 increases MYC translation by m^6^A methylation [44]. Embryonic ectoderm development protein (EED) increases METTL3 by methylating miR-338-5p. METTL3 increases oncogene CDCP1 [50], thus promoting GC cells proliferation, migration and invasion. (Figure 2C).

### 2.4. Pancreatic Cancer

Pancreatic cancer is a tumor of the digestive tract with lethal malignancy and poor prognosis with 5-year survival rates at 9% [37]. METTL3 expression and RNA m^6^A modification is higher in pancreatic cancer tissues compared to normal tissues and high METTL3 expression is related to high pathological stage and high lymph node metastasis. Conversely, METTL3 knockdown promoted pancreatic cancer cell proliferation, invasion, and migration in vitro [51]. Since cigarette smoking is linked to pancreatic cancer, a study found that cigarette smoke condensate can increase expression of METTL3 by causing DNA hypomethylation of the METTL3 promoter, thus allowing the transcription factor NFIC to activate METTL3 transcription [52]. Then, METTL3 promotes the maturation of miR-25-3p mediated by NF-κB associated protein (NKAP), which leads to increased carcinogenicity in pancreatic ductal epithelial cells [52]. High levels of miR-25-3p in smokers and in pancreatic cancer tissues are associated with poor prognosis in pancreatic cancer patients [52] (Figure 2D). miR-25-3p inhibits PHLPP2, thereby activating the oncogenic AKT-p70S6K signaling pathway, which induces malignant phenotypes of pancreatic cancer [52]. METTL3-depleted pancreatic cancer cells show higher sensitivity to anticancer reagents and irradiation. This means that METTL3 is probably a potential target for increasing therapeutic effect in patients with pancreatic cancer [53].

### 2.5. Hepatocellular Carcinoma (HCC) and Hepatoblastoma

Hepatocellular carcinoma (HCC) ranks as the sixth common malignancy worldwide and has higher mortality among patients [54]. METTL3 is expressed at higher levels in tumor samples compared to normal liver cells and higher expression of METTL3 is correlated with worse survival outcomes in HCC patients [55,56,57]. METTL3 knockdown of HEPG2 and Huh7 cells slows cell proliferation, reduces colony formation, suppresses cell migration and impairs in vivo tumor formation [57]. Mechanistically, METTL3 methylates the tumor suppressor SOCS2 and its mRNA is degraded in a YTHDF2-dependent manner, thus contributing to tumorigenesis [57]. In another study, METTL3 was found to methylate and stabilize *LINC00958* lncRNA leading to its upregulation [56]. *LINC00958* exerts oncogenic properties in HCC by downregulating miR-3619-5p to upregulate hepatoma-derived growth factor (HDGF) expression, leading to reprogramming of lipid metabolism to promote HCC [56]. METTL3 is also involved in elevating mTORC1 activity and glycolysis, thus contributing to tumor progression of HCC cell lines [55]. METTL3 also plays a role in sorafenib sensitivity in HCC as METTL3 knockdown cells exhibited a two-fold increase in the IC_50_ of sorafenib compared to METTL3 expressing cells [58]. Interestingly, METTL3 was also shown to suppress autophagy in HCC cells by methylating FOXO3 mRNA, leading to its upregulation in a YTHDF1-dependent manner [58]. This study identifies METTL3 and FOXO3 as critical regulators of autophagy-induced sorafenib resistance [58].

The m^6^A regulators METTL3, WTAP, FTO and YTHDF2 are elevated in tumor vs control tissues from hepatoblastoma patients and all four regulators contributed to the proliferation and colony formation of HepG2 cells [59]. This study chose to focus on the role of METTL3 due to elevated m^6^A levels in tumor vs normal patient total RNA samples although this was measured in only five patient samples. METTL3 knockdown decreased CTNNB1 mRNA methylation level and stability to inhibit Wnt/β-catenin signaling pathway in Huh6 and HepG2 cells [59]. In another study, METTL3 was identified as a direct target of microRNA-186, which is weakly expressed in hepatoblastoma tissue [60]. In addition, overexpression of miR-186 significantly inhibited the invasive phenotype in vitro and in vivo, which could be reversed by METTL3 overexpression. This study also corroborates with the previously mentioned study by demonstrating that the miR-186/METTL3 axis regulates the Wnt/β-catenin signaling pathway, contributing to tumorigenesis [60] (Figure 2E).

### 2.6. Colorectal Carcinoma (CRC)

METTL3 shows oncogenic effects in promoting the proliferation and metastasis of CRC using in vitro and in vivo models and could also act as a potential biomarker for CRC prognosis [61]. CRC metastasis tissues have high expression of METTL3 and elevated METTL3 correlates with poor prognosis [62]. Down regulation of METTL3 significantly inhibits the self-renewal and migration of CRC stem cells in vitro [62]. MeRIP-seq results showed that SOX2 is the downstream target gene of METTL3. Since SOX2 is an important transcription factor for maintaining self-renewal and proliferation of pluripotent stem cells, it plays an important part in the development of malignant tumors. The coding region of methylated SOX2 mRNA can be recognized by IGF2BP2 to inhibit the degradation of SOX2 mRNA. Supporting this, the expression of SOX2 target genes is positively correlated with METTL3 and IGF2BP2 in CRC [62]. In a separate study, METTL3 promotes activation of the glycolytic pathway in CRC patients by interacting directly with the 5′/3′UTR region of HK2 and the 3′UTR region of GLUT1. HK2 and GLUT1 transcripts are then stabilized by the m^6^A reader IGF2BP2 [61]. GLUT1 promotes glucose uptake and lactate production, which causes mTORC1 signaling activation and CRC development [63]. METTL3 and its pathway are potential therapeutic targets for CRC patients with high glucose metabolism [63]. Xiang et al. found that METTL3 promoted CRC progression through enhancing MYC expression in an IGF2BP1-dependent manner [64]. Another study demonstrated a link between the gut microbiome and cellular m^6^A levels by showing that butyrate, a bacterial short-chain fatty acid, decreases METTL3 levels in vitro, thus suppressing tumor cell growth [65]. Further elucidation of this mechanism of microbiome-mediated tumor suppression through m^6^A regulation would be an interesting avenue of research [65] (Figure 2F).

### 2.7. Breast Cancer

Breast cancer is the most common cancer in females in the USA [37]. Upregulation of METTL3 is observed in breast cancer tissues and cells and its elevated expression is correlated with worse patient survival [66]. Since breast cancer is a heterogenous disease divided into various molecular subtypes, METTL3 expression is found to be higher in normal-like and Luminal A/B subtypes compared to triple negative and HER2 positive subtypes [67]. Knockdown of METTL3 in Luminal A and triple negative cell lines can reduce methylation levels, reduce tumor cell proliferation, accelerate apoptosis and inhibit tumor growth in a BALB/c xenograft model [66]. Bcl-2 transcripts are upregulated following METTL3 methylation, indicating that METTL3 may promote breast cancer proliferation by regulating apoptosis [66]. Another study found that METTL3 expression in breast cancer tissues is positively correlated with HBXIP expression and HBXIP is indeed a target of METTL3 methylation [68]. HBXIP upregulates METTL3 by inhibiting miRNA let-7g, which downregulates METTL3 expression [68]. The positive feedback loop of HBXIP/let-7g/METTL3/HBXIP promotes the proliferation of breast cancer cells [68]. In one study, by utilizing a genetically defined immortalized and oncogenic transformed human mammary epithelial cell (HMEC) model, METTL3 expression was downregulated and m^6^A levels on mRNA were lower in immortalized and transformed HMECs compared to primary, untransformed HMECs [69]. Given the nature of this phenotype, the authors surprisingly found that overexpression of METTL3 and METTL14 in transformed cells but not immortalized cells can promote cell proliferation and migration [69]. These results show that the function of METTL3 in this model is nuanced, possibly playing different roles in primary HMECs vs transformed or immortalized HMECs [69]. Tamoxifen contributes to treating estrogen receptor (ER)-positive breast cancer. In tamoxifen-resistant MCF-7 cells, increased METTL3 confers a greater resistance to tamoxifen by promoting AK4 expression. AK4 inhibits mitochondrial apoptosis and promotes ROS production [70]. Another study found that METTL3 decreases Collagen type III alpha 1 chain (COL3A1), thus inhibiting triple-negative breast cancer (TNBC) cell metastasis [71]. The numerous genetic mutations present in breast cancers and the various molecular subtypes warrants a more in-depth analysis of the role of METTL3 and m^6^A in breast cancer (Figure 2G).

### 2.8. Ovarian Cancer

Using immunohistochemistry on 52 patient samples, in one study METTL3 was highly expressed in ovarian cancer tissues compared to adjacent normal tissues, and was significantly correlated to tumor grade [72]. METTL3 knockdown in SKOV3 and OVCAR3 cells caused decreased cell proliferation, increased apoptosis, and perturbed migration and invasion in Transwell assays due to lower levels of phosphorylated Akt [72]. In another study using a larger cohort of 162 ovarian carcinoma samples, METTL3 expression was positively correlated with tumor histological grades pT, pN, pM and FIGO stage, suggesting that METTL3 is important for metastasis of ovarian cancer [73]. This study further shows that METTL3 can promote AXL translation and the EMT process [73]. METTL3 accelerated the maturation of miR-126-5p via the m^6^A modification of pri-miR-126-5p. miR-126-5p can activate the PI3K/Akt/mTOR pathway by targeting PTEN, thus facilitating ovarian cancer cell proliferation, migration, and invasion [74]. These studies demonstrate the feasibility of METTL3 as a new prognostic indicator and therapeutic target in ovarian cancer (Figure 2H).

### 2.9. Cervical Cancer

METTL3 is independent indicator for poor prognosis in early stage cervical cancer patients [75]. METTL3 targets the 3′-UTR of hexokinase 2 (HK2) mRNA to enhance HK2 stability in a YTHDF1-dependent manner, which promotes the proliferation and aerobic glycolysis of cervical cancer cells [76]. Hu et al. found that METTL3 increased the mRNA stability of RAB2B in an IGF2BP3-dependent manner, which can facilitate cervical cancer development [77]. These studies suggest that METTL3 might be a potential target for cervical cancer therapy (Figure 2H).

### 2.10. Glioblastoma (GBM)

Glioma stem-like cells (GSCs) contribute to poor prognosis as they are responsible for the high rate of recurrence and chemotherapy resistance in GBM [78]. Since METTL3 has been shown to play a role in regulating stem cell pluripotency and differentiation [79], its role in GSCs has been studied. METTL3 plays a crucial role in the maintenance and dedifferentiation of GSCs [78,80]. Knockdown of METTL3 in GSCs promotes growth and self-renewal in vitro and sphere formation [80]. The expression of several oncogenes including ADAM metallopeptidase domain 19 (ADAM19), EPH receptor A3 (EPHA3) and Kruppel like factor 4 (KLF4) are elevated in METTL3 and METTL14 knockdown GSCs [80,81]. Conversely, a separate study suggested that METTL3 was upregulated in GBM tumors and its knockdown inhibited tumor growth in U87/shMETTL3-injected mice. Further research suggested that SOX2 stabilized by METTL3 with recruitment of HuR was related to the maintenance and radiation resistance of glioma stem-like cells [78]. Furthermore, METTL3 promotes the temozolomide (TMZ) resistance of glioma cells by increasing DNA repair genes O^6^-methylguanine-DNA methyltransferase (MGMT) and alkylpurine–DNA–N-glycosylase (ANPG) [82]. GSEA analysis of METTL3 knockdown vs control GSCs identified reduction of Notch signaling, a pathway promoting keratinocytes differentiation and inhibiting tumorigenesis including NOTCH1, NOTCH3, NOTCH4, and HES1. METTL3 knockdown downregulated NOTCH1 and HES1 protein expression [83]. METTL3 promotes cell proliferation dependent on YTHDF1 by targeting RNA-binding protein adenosine deaminases acting on RNA-1 (ADAR1) which catalyzes A-to-I RNA editing. Furthermore, ADAR1 stabilizes cyclin-dependent kinase 2 (CDK2) mRNA, which is an important cell cycle kinase that promotes cell proliferation. Interestingly, A-to-I RNA editing is decreased in GBM, because METTL3 boosts the amount of transcripts depositing m^6^A, inhibiting editing at multiple sites [84] (Figure 2I).

### 2.11. Renal Cell Carcinoma (RCC)

Renal cell carcinoma (RCC) is a frequent malignant tumor of the adult kidney [54]. Samples from a cohort of 145 patients found that high expression of METTL3 correlated with longer patient survival, indicating a tumor suppressive role for METTL3 in RCC [85]. Consistent with the clinical findings, METTL3 was expressed at lower levels in CAKI-1, CAKI-2, and ACHN RCC cell lines compared to a normal human renal tubular epithelial cell line, HK-2 [85]. In CAKI-1 and CAKI-2 cells, METTL3 was found to slow the proliferation, colony formation, migration, and invasion of RCC cells [85]. METTL3 mediates RCC cell proliferation possibly by negative regulation of the PI3K-Akt-mTOR pathway. In a recent TCGA analysis of kidney renal papillary cell carcinoma (KIRP) (n = 289, normal samples = 32), there was no significant different expression of METTL3, WTAP, RBM15, FTO YTHDC2 and YTHDC1 in KIRP compared to normal kidney tissue samples [86]. In another study, METTL14 decreased P2RX6 (ligand-gated ion channel receptors) expression activation-facilitated RCC cell migration and invasion by inhibiting ATP-induced Ca^2+^ influx to increase the ERK1/2 and MMP9 signaling pathway in SN12-PM6 and 786-O RCC cell lines compared to HK2 [87] (Figure 2J). In RCC, it is not known whether the methylation-dependent or methylation-independent mechanisms of METTL3 contribute to tumorigenesis. Given the controversial findings of these studies, more in-depth studies that experimentally determine the direct mRNA targets of METTL3 should be done to clarify the role of METTL3 in RCC.

### 2.12. Bladder Cancer

Compared with the paracancerous bladder urothelial tissue, the expression of METTL3 in the bladder cancer tissue is increased. Knockdown of METTL3 can significantly inhibit the proliferation, colony formation, migration and invasion of bladder cancer cells in vitro [88]. Using nude mice models, METTL3-depleted UM-UC-3 cells produced smaller tumors compared to control cells and had reduced metastasis [88]. METTL3 methylates the mRNA of the tumor suppressors SETD7 and KLF4, which are then degraded by YTHDF2 to promote the development of bladder cancer [88]. In a chemically induced bladder cancer model of normal human uroepithelial SV-HUC-1 cells and prostate epithelial RWPE-1 cells, METTL3 can upregulate CUB domain-containing protein 1 (CDCP1) in bladder cancer tissues using both methylation-dependent and methylation-independent mechanisms of METTL3 [89]. Inhibition of the METTL3-m^6^A-CDCP1 axis can slow down the growth and progression of bladder cancer cells [89]. This axis can also promote the malignant transformation of urothelial cells and the occurrence of bladder cancer in vivo and in vitro [89]. METTL3 can also promote the progression of bladder cancer through both the NF-κB and MYC signaling networks since AFF4, IKBKB, and p65 as well as MYC transcripts are methylated and stabilized by METTL3 [90]. METTL3 can enhance cell adhesion by upregulating ITGA6, which is linked to poor prognosis in bladder cancer [91]. Since ITGA6 enhances the growth and metastasis of bladder cancer cells, METTL3 knockout leads to reduced adhesion, proliferation, migration and invasion of bladder cancer cells [91]. METTL3 can downregulate PTEN expression by interacting with the microprocessor protein DGCR8 and positively regulating pri-miR221/222 processing in an m^6^A-dependent manner, thus exerting oncogenic effects in bladder cancer [92]. These results collectively suggest that METTL3 plays an important role in the progression of bladder cancer (Figure 2J).

### 2.13. Prostate Cancer

METTL3 is upregulated in prostate cancer cells compared to normal cells and is a poor prognostic factor for patient survival [93,94]. Genetic depletion and overexpression of METTL3 in various prostate cancer cell lines demonstrate that METTL3 can significantly promote the proliferation, migration and invasion of prostate cancer cells [93,94]. In terms of mechanisms, one study revealed that METTL3 can elevate the expression of MYC mRNA and protein levels by increasing the m^6^A level of the MYC transcript, which leads to carcinogenesis of prostate cancer [94]. Another study showed that METTL3 can methylate LEF1 mRNA leading to increased LEF1 protein expression. Since LEF1 is a positive regulator of the Wnt signaling pathway, METTL3 can promote prostate cancer progression through the METTL3-LEF1-Wnt axis [93]. METTL3 can also positively regulate the sonic hedgehog (SHH) pathway component GLI1, which is a transcription factor that activates c-Myc and cyclin D expression [95]. Li et al. showed that YTHDF2 degraded the tumor suppressors LHPP and NKX3-1 mRNA in a METTL3-m^6^A-dependent manner to regulate AKT phosphorylation-induced prostate tumor progression [96]. Another study found that METTL3 advanced the expression of Integrin β1 (ITGB1) in a m^6^A-HuR-dependent manner, promoting the bone metastasis of prostate cancer cells [97] (Figure 2J).

### 2.14. Melanoma

Studies have shown that melanoma cell lines express higher levels of METTL3 than normal melanocytes, resulting in increased m^6^A methylation on total RNA [98]. shRNA-mediated knockdown of METTL3 attenuates melanoma cell colony formation and invasion in vitro [98]. In A375 and WM793 cells, METTL3 regulated matrix metalloprotein-2 (MMP2) and N-cadherin protein levels, respectively [98]. Uveal melanoma (UM) is the most common intraocular tumor with high morbidity because of frequent metastasis [99]. METTL3 and METTL14 are significantly upregulated in UM cell lines compared to normal uveal cells [99]. METTL3 knockdown in UM cell lines decreases proliferation, colony formation, migration, and invasion by decreasing c-Met expression in a methylation dependent manner [99]. Since c-Met is upstream of the Akt pathway, METTL3 knockdown also decreases total Akt levels, indicating that the METTL3/c-Met/Akt axis is important for melanoma cell survival and migration. METTL3 inhibitor, cycloleucine, also shows similar effects to METTL3 knockdown cells, providing new insight for the treatment of UM [99] (Figure 2K).

## 3. METTL3 Functions in Tumor Immune Microenvironment

Though immunotherapy with programmed cell death-1 (PD-1) checkpoint blockade has achieved great progress in many types of cancers, a subset of cancer types still shows less or no response. Increasing evidence is depicting the roles of METTL3 in the tumor immune microenvironment and METTL3 as a potential therapeutic target for tumor immunotherapy.

**Figure 3:**
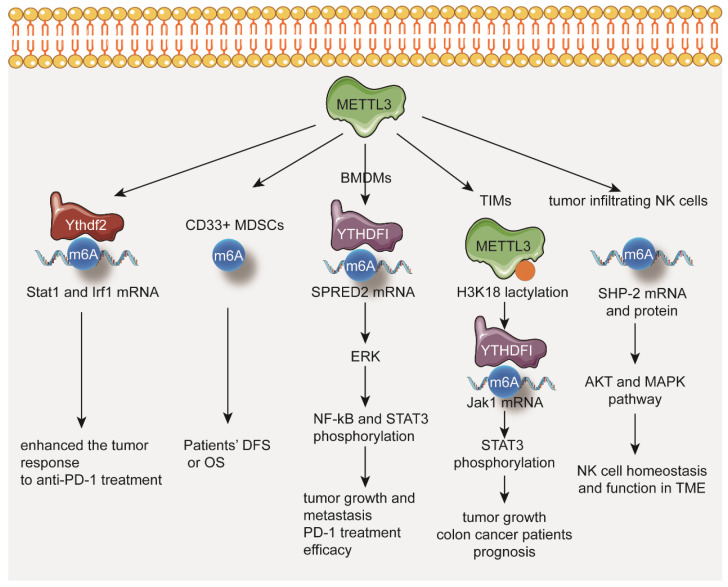
METTL3 in regulation of tumor immune microenvironment.

In mismatch-repair-proficient or microsatellite instability-low (pMMR-MSI-L) CRC and melanoma mouse models, depletion of METTL3 enhances the tumor response to anti-PD-1 treatment through stabilizing the Stat1 and Irf1 mRNA via YTHDF2 [100] (Figure 3).

In cervical cancer patients, the levels of METTL3 and CD33^+^ MDSCs in tumor tissues are significantly associated with reduced DFS or OS [101] (Figure 3).

In METTL3-deficient bone marrow-derived macrophages (BMDMs), the expression of M1-associated genes (Tnf-α and Il-6) and M2-associated genes (Arg1) is significantly increased. Loss of METTL3 impairs the YTHDF1-mediated translation of SPRED2, which increases the phosphorylation of NF-kB and STAT3 through the ERK pathway. Depletion of METTL3 in myeloid cells promotes tumor growth and metastasis. Furthermore, the efficacy of PD-1 checkpoint blockade is impaired in METTL3-deficient mice [102] (Figure 3).

Increased expression of METTL3 in tumor infiltrating myeloid cells (TIMs) correlates with the poor prognosis of colon cancer patients. Increasing lactate in tumor microenvironment induces METTL3 upregulation in TIMs via H3K18 lactylation. Depletion of METTL3 in myeloid cells decreases tumor growth in mice. METTL3 mediates m^6^A modification on Jak1 mRNA in TIMs and enhances JAK1 protein translation through the m^6^A-YTHDF1 axis, subsequently increasing phosphorylation of STAT3 (Figure 3).

A positive correlation between protein expression levels of METTL3 and effector molecules in tumor infiltrating NK cells has also been found. NK-cell-specific deletion of METTL3 in mice disrupts NK cell infiltration and function in the tumor microenvironment, leading to tumor progression. Mechanistically, METTL3 regulates SHP-2 m^6^A modification which subsequently stabilizes SHP-2 protein expression in NK cells. In METTL3-deficient NK cells, reduced SHP-2 activity suppresses the activation of the AKT and MAPK from IL-15 signaling (Figure 3).

These findings indicate that METTL3-mediated m^6^A methylation plays important functions in the tumor immune microenvironment.

## 4. Conclusions and Perspectives

Cancer cells utilize METTL3 to exploit specific molecular pathways to favor proliferation, metastasis, invasion, drug resistance, and the maintenance of cancer progenitor cells (see Table 1). Targeting m^6^A modifying enzymes such as METTL3 or METTL14 by selective inhibitors represents a promising state-of-the-art therapeutic strategy to treat cancers as it acts upstream of notable oncogenic pathways such as MYC, Akt and SHH. MYC is one of the most sought-after drug targets and developing specific MYC inhibitors has proved challenging. Since METTL3 is an upstream regulator of MYC in a number of cancers (AML, bladder cancer, and prostate cancer), targeting METTL3 can modulate this historically difficult drug target. Presently, multiple METTL3 specific inhibitors are already in various stages of development [103]. STM2457, a highly selective catalyst inhibitor of METTL3, results in decreased m^6^A levels of leukemogenic mRNAs, causing decreased growth, increased differentiation and apoptosis in a mouse AML model [104].

In addition, mRNA m^6^A methylation is interrelated with cellular immunity. Han, D et al. found that loss of YTHDF1 strengthened the function of dendritic cells tumor antigen cross-presentation and CD8^+^ T response to tumor [105]. Recent research has showed that FTO enhanced melanoma tumorigenesis and PD-1 antibody treatment controlled melanoma development in FTO knockdown tumors on the complete immunity system [106]. The significant investment and research into therapeutics targeting METTL3 underscores the novelty and promise of targeting mRNA modifications in cancer.

## Figures and Tables

**Table 1:** Expression, role, biological performance and molecular mechanisms of METTL3 in various cancers.

Cancer Type	Expression	Role	Biological Performance	Molecular Mechanism	Ref.
Acute myeloid leukemia	Upregulated	Oncogene	Stem cell differentiation, proliferation, cell cycle, tumorigenesis	c-MYC, MYB, PTEN, BCL2, SP1 and SP2, ect	[8,36]
Lung cancer	Upregulated	Oncogene	Survival, proliferation, migration, invasion, metastasis, angiogenesis	EGFR, TAZ, DNMT3A, miR-1246/PEG3, MALAT1, JUNB, VASH1, ZBTB4/EZH2, ect	[30,39,40,41,42,43]
Gastric cancer	Upregulated	Oncogene	Proliferation, migration, invasion, metastasis	HDGF, ZMYM1, BATF2, CDCP1, ect	[46,47,49,50]
Pancreatic cancer	Upregulated	Oncogene	Proliferation, migration, invasion, chemoresistance	miR-25-3p/PHLPP2/ AKT-p70S6K, ect	[51,52]
Hepatocellular carcinoma and hepatoblastoma	Upregulated	Oncogene	Proliferation, colony formation, migration, tumorigenesis	SOCS2, HDGF, mTORC1, FOXO3, CTNNB1/Wnt/β-catenin, ect	[55,56,57,58,59]
Colorectal carcinoma	Upregulated	Oncogene	Self-renewal, proliferation, metastasis, tumorigenesis	SOX2, HK2, GLUT1, MYC, ect	[61,62,63]
Breast cancer	Upregulated	Oncogene	Proliferation, apoptosis, migration	Bcl2, HBXIP, AK4, ect	[66,68,70]
Ovarian cancer	Upregulated	Oncogene	Proliferation, migration, invasion, metastasis	AXL, PTEN, ect	[73,74]
Cervical cancer	Upregulated	Oncogene	Proliferation, aerobic glycolysis	HK2, RAB2B, ect	[76,77]
Glioblastoma	---	Tumor suppressor	Self-renewal, stem cell differentiation	ADAM19, EPHA3, KLF4, ect	[80,81]
	Upregulated	Oncogene	Proliferation	SOX2, ADAR1/CDK2, ect	[78,84]
Renal cell carcinoma	Downregulated	Tumor suppressor	Proliferation, colony formation, migration, invasion	PI3K/Akt/mTOR, P2RX6, ect	[85,86,87]
Bladder cancer	Upregulated	Oncogene	Proliferation, colony formation, migration invasion	SETD7, KLF4, CDCP1, MYC, ITGA6, ect	[88,89,90,91]
Prostate cancer	Upregulated	Oncogene	Proliferation, migration, invasion, tumorigenesis	MYC, LEF1/Wnt, GLI1, LHPP, NKX3-1, ITGB1, ect	[93,94,95,96,97]
Melanoma	Upregulated	Oncogene	Colony formation, invasion	MMP2, N-cadherin, c-Met, ect	[98,99]

## Data Availability

Not applicable.

## Abbreviations

METTL3	Methyltransferase-like 3
YTHDF1	YTH *N*^6^-Methyladenosine RNA Binding Protein 1
YTHDF2	YTH *N*^6^-Methyladenosine RNA Binding Protein 2
YTHDC2	YTH Domain Containing 2
AML	Acute myeloid leukemia
HCC	Hepatocellular carcinoma
CRC	Colorectal carcinoma
RCC	Renal cell carcinoma
